# A cautionary tale for AI and machine learning in psychiatry

**DOI:** 10.1038/s41398-026-03930-w

**Published:** 2026-03-08

**Authors:** Zhe Sage Chen, Katharina Schultebraucks, Wei Wu

**Affiliations:** 1 Department of Psychiatry, New York University Grossman School of Medicine, New York, NY 10016 USA; 2 Department of Neuroscience, Institute of Translational Neuroscience, New York University Grossman School of Medicine, New York, NY 10016 USA; 3 Department of Biomedical Engineering, New York University Tandon School of Engineering, Brooklyn, NY 11201 USA; 4 Department of Population Health, New York University Grossman School of Medicine, New York, NY 10016 USA; 5 School of Medicine, Shanghai Jiaotong University, Shanghai, China

**Keywords:** Biomarkers, Psychiatric disorders

## Abstract

Artificial intelligence (AI) and machine learning (ML) have seen remarkable growth in mental health applications over the past few decades, demonstrating significant potential to transform psychiatric care. Despite these advancements, the translation of AI systems into clinical practice remains fraught with challenges. This Perspective examines critical hurdles in psychiatric AI research, emphasizing limitations in research rigor, model reliability, interpretability, clinical utility, and ethical considerations. We argue that a human-assisted AI framework—incorporating incremental feedback, self-adaptation, and dynamic collaboration—can address biases, enhance transparency, and build trust in AI systems. Moreover, initiatives in clinical education, cultural adaptation, and data/software sharing are essential to fostering public engagement, data transparency, and research reproducibility. By focusing on these areas, we aim to bridge the gap between AI potential and its successful, ethical implementation in mental health care, guiding the development of trustworthy, effective, and culturally adaptive AI-powered psychiatric tools.

## Introduction

The rising cost of mental health and substance abuse places a significant burden on healthcare systems and the global economy. Nearly 20% of adults in the United States live with mental illness, while the increasing demand for psychiatric care continues to overwhelm an already strained mental health system [1]. Recent advancements in AI and ML (Box 1) have demonstrated significant successes in medicine, such as optimizing treatment strategies and predicting readmission outcome [2, 3]. This raises an important question: can AI reliably assist psychiatrists in addressing the mental health crisis?

The debate over the pros and cons of AI in psychiatric research persists [4]. While supporters highlight AI’s potential for scalability, accessibility, and generalizability, others remain cautious about its limitations, particularly in precision psychiatry [5–9]. The advent of large language models (LLMs), such as ChatGPT, has revolutionized human-computer interaction, showcasing physician-level diagnostic capabilities and human-like empathy [3, 10]. However, tangible breakthroughs in mental health care remain limited, prompting valid reservations [11–13]. For instance, a 2019 survey of 800 psychiatrists across 22 countries found that only 4% believed AI could ever replace psychiatrists in complex psychiatric tasks, though many see its potential as an assistive tool [14].

Traditional methods for diagnosing and managing psychiatric disorders—such as clinical evaluations and questionnaires—are time-consuming and often lack precision [15]. Precision psychiatry, on the other hand, offers a more individualized approach to diagnosis, prognosis, and treatment by leveraging clinical symptoms, biological markers, and patient histories. Emerging technologies in neuroimaging, wearable devices, and social media applications have opened new doors for AI-powered digital phenotyping and a transformative shift in psychiatric care [16].

In this Perspective, we review recent advances in AI and explore the technical challenges and clinical barriers to integrating AI into mental health practice. Additionally, we offer actionable recommendations to maximize the clinical viability of AI and propose a human-assisted AI framework to enhance interpretability, reliability, and trust in AI-based clinical decision-making.

Box 1 **Glossary of key terms** **Artificial Intelligence (AI):** AI is the overarching field concerned with creating systems capable of performing tasks that typically require human intelligence, such as perception, reasoning, problem-solving, and decision-making. In medicine, AI encompasses a spectrum of approaches—from rule-based expert systems to data-driven predictive models.

**Machine Learning (ML):** ML is a subset of AI that enables systems to learn patterns from data and improve performance (such as prediction and classification) without explicit rule programming. Algorithms such as Logistic Regression, Random Forests, XGBoost (eXtreme Gradient Boosting), and Support Vector Machines (SVMs) have been widely applied in psychiatry to predict treatment outcomes, relapse risk, and diagnostic classification based on clinical and neuroimaging data. Deep learning (DL) is a specialized branch of ML that employs multilayered artificial neural networks (ANNs) to extract complex, hierarchical representations from large datasets. It has achieved successes in analyzing high-dimensional neuroimaging, multimedia and behavioral data, though at the cost of interpretability.

**Large Language Models (LLM):** LLMs are advanced DL architectures trained on massive text corpora to understand, summarize, and generate human-like language. In psychiatry, LLMs hold promise for clinical documentation, natural language analysis of patient narratives, and decision support. However, their integration requires careful attention to issues of bias, transparency, and clinical validation.

**Biomarker** is an objectively measurable indicator of a biological process, pathogenic state, or therapeutic response. It does not need to be a biological molecule itself, but rather any quantifiable feature—such as a neural signal, genetic variant, or behavioral metric—that reliably reflects an underlying biological condition. Major categories of biomarkers, as defined by FDA and NIH’s Biomarkers Definitions Working Group, include *diagnostic biomarkers, prognostic biomarkers, predictive biomarkers, monitoring biomarkers, response biomarkers*, and *safety biomarkers*.

## A partnership beween AI and psychiatry and where we stand

Development of evidence-based, data-intensive AI algorithms that support decision-making in psychiatry has been a long-standing holy grail. Specifically, AI tools are showing real promise in suicide risk prediction, identifying individual at high risk much earlier and more reliably, especially when combining multiple sources of data. For example, an ensemble ML strategy was used to predict suicide attempts in 1818 patients after an emergency department visit. This model used a mix of patient self-report, electronic health record (EHR) data, and clinician assessment, and achieved better accuracy than clinicians alone: an AUC of ~0.77 for one-month prediction and ~0.79 for six-month prediction when all inputs were combined [17]. Another promising direction is using user-generated text (e.g. diary entries or app-based logs) processed by LLMs. A recent study has shown that using diaries and LLMs to detect depression achieved accuracies of ~90% in distinguishing depressed vs non-depressed participants, with high specificity [18]. These examples suggest that combining subjective, behavioral, and biological data (text, imaging, EHRs) yields the best performance, even though many applications are still early stage and need further validation.

On the other hand, the complexity and heterogeneity of mental illnesses remains a challenge in AI/ML-driven diagnosis for large sample sizes. For example, in terms of diagnosis and treatment of mood disorders like depression, the results are mixed but informative. A large neuroimaging-based study (n = 1801 patients) attempted to define a biomarker signature for major depressive disorder (MDD) using machine learning across many brain imaging modalities. While the classifier did better than chance, its accuracy was only about 62%, which is insufficient for clinical use in isolation [19]. Another recent study has critically evaluated the generalizability of ML models in predicting treatment outcomes in schizophrenia across multiple clinical trials. Surprisingly, the model accuracy significantly declined to a chance level when applied to independent clinical trials, suggesting that there is a critical need for validating clinical prediction models across diverse clinical samples [20].

We recognize that current AI-powered psychiatric research still faces significant technical and practical obstacles that merit critical evaluation. Some challenges are broad and apply across data science applications, such as data and algorithmic biases, low data quality, and imbalanced samples. Others are deeply tied to the methodology (e.g., interpretability issues) or influenced by policy and clinical culture, including concerns around AI ethics and regulation. It is equally important to set realistic goals for AI in psychiatry, along with clear milestones to measure progress. Without aligned expectations, differing views of what constitutes “success” may emerge. Achieving a successful partnership between AI and psychiatry requires a cohesive, well-structured foundation — yet there are countless small ways such efforts can falter. In the paragraphs that follow, we revisit and elaborate on several major critiques to provide a roadmap for addressing these pressing issues.

### Critique #1: Biased data can be harmful

AI in mental health relies on data, but data are inherently limited and prone to biases, such as gender, racial, and age bias, that can arise during psychiatric practice and data collection [21]. Physicians’ cognitive biases, flawed study designs, and evolving data contribute to these issues. Dynamic bias assessment is crucial, as AI models must adapt to new data to remain reliable and fair. Without this, biases in data and algorithms may persist or amplify, leading to significant risks like overdiagnosis, overtreatment, or flawed public policy.

One source of bias stems from under-reporting and under-coding, particularly in patients from low socioeconomic backgrounds who lack medical access, leading to data gaps [22]. Self-reported psychiatric data, common in mental health research and AI-dependent apps, also introduces bias due to factors like recall errors and social desirability [23]. Clinicians are advised against using digital biomarkers unless models ensure equitable predictions across diverse groups.

Additionally, crystallization of bias in data occurs when skewed, incomplete, or unrepresentative datasets are used to train AI models, causing those biases to become fixed and amplified in predictive outcomes. Sampling and domain biases are not uncommon in psychiatric data.

Labeling accuracy further affects data quality. Diagnosing mental illness, unlike physical conditions, remains challenging due to overlapping symptoms, cultural differences, and heterogeneous presentations [24]. Poorly labeled data can create significant biases, particularly in transdiagnostic frameworks attempting to redefine psychiatric classifications [25]. Without robust data validation, biased results can hinder AI’s reliability in mental health. Unproven AI claims must be approached cautiously to ensure data quality and fairness.

### Critique #2: Hurdles in psychiatric biomarker discovery and biological-clinical alignment

A key goal of AI in psychiatry is the discovery of biomarkers (Box 1) for diagnosis, prognosis, and treatment prediction using multimodal data (e.g., genetic, neuroimaging, behavioral) [26, 27]. Although AI excels at uncovering complex associations, numerous conceptual and technical challenges hinder progress [28]. While many studies propose potential biomarkers, few are *clinically viable* due to issues with generalizability and context-dependence, as seen in schizophrenia biomarker research that failed across independent clinical trials^20^. AI-driven biomarker discovery is often hampered by an overreliance on observational hypotheses and insufficient focus on mechanistic underpinnings. Furthermore, study designs frequently fail to consider validation requirements, such as constructing negative classes to assess sensitivity and specificity. These limitations raise concerns about the robustness of AI models in psychiatric research.

**Fig. 1:**
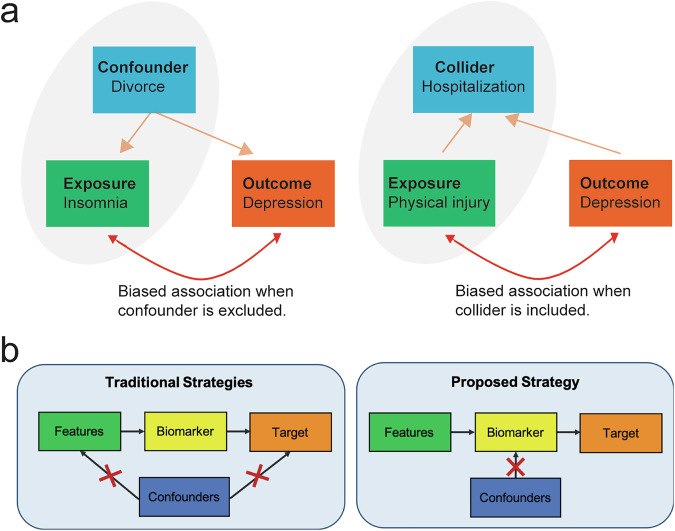
Development of causal inference tools for precision psychiatry. **a** Illustration of confounding and collider biases in predictive diagnosis in psychiatry. Confounding occurs when a common cause exists for both exposure and outcome, where a collider is a common effect of both exposure and outcome. Excluding a confounder or including a collider in a predictive model can lead to biased associations. **b** The confounders are traditionally controlled for by eliminating their influences on either the features or the target variable. However, these strategies do not integrate confounders into the modeling procedure. To address this limitation, an improved strategy is to decorrelate the biomarker and target at the biomarker optimization stage. For instance, additional constraints can be imposed on the biomarker such that it is correlated with the confounders. This strategy can be readily applied to the existing machine learning algorithms.

From a data perspective, mental disorders often involve multifaceted biological and molecular alterations; however, aggregating multimodal datasets and addressing missing data remain significant hurdles. Current statistical methods often assume missing data are random—a flawed assumption that complicates biomarker validation [29]. Analytical variability further deepens the challenge, as different preprocessing steps produce inconsistent results [30], and confounders like sex, age and socioeconomic status obscure causal relationships (Fig. 1). Existing methods (e.g., matching, stratification) mitigate confounder-induced bias but often reduce statistical power and require post-hoc checks [31, 32]. Principled strategies specifically designed for identifying unconfounded biomarkers are yet to be developed (Box 2) [33–35].

Clinical and biological heterogeneity further complicate biomarker development. Biological variations reflect differences in disease mechanisms, while clinical heterogeneity encompasses variations in symptom presentation, treatment response, and diagnostic outcomes [36]. High-dimensional biological data are abundant but often poorly aligned with treatment outcomes due to limited clinical datasets. Unsupervised ML can parse biological heterogeneity, but without clear integration of clinical data, resulting biomarkers lack predictive utility [37–40]. This biological-clinical data misalignment underscores the need for innovative methods to merge rich cross-sectional biological data with labeled longitudinal clinical outcomes, adhering to the established frameworks like NIMH’s Research Domain Criteria (RDoC), WHO’s ICD-11, and American Psychiatric Association’s DSM-5-TR. Overall, generalizable biomarkers require new strategies that address study design flaws, confounders, multimodal data integration, and the alignment of biological and clinical heterogeneity. The development of principled AI methods remains crucial to overcoming these barriers in psychiatric research.

Box 2 **Causal inference in precision psychiatry** Causal inference can be used to account for confounders in biomarker discovery. As a simple illustration of the confounder effect in predictive modeling, we may adapt a standard linear predictive modeling framework to infer unconfounded biomarkers. By imposing additional constraints on the biomarkers such that they are uncorrelated with the confounders, the predictive framework can be formulated as a convex optimization problem:

$$\mathop{\min }\limits_{{\bf{w}}}{\Vert {\bf{X}}{\bf{w}}-{\bf{y}}\Vert }_{2}^{2}+\lambda {\Vert {\bf{w}}\Vert }_{2},$$

where **X** represents the data matrix with rows corresponding to samples and columns corresponding to input features, **y** represents the target vector containing the outcomes across samples, **w** is the unknown regression weight vector, and $\lambda$ is the regularization parameter. As an extension, an unconfounded version of ridge regression can be cast as a constrained optimization problem:

$$\mathop{\min }\limits_{{\bf{w}}}{\Vert {\bf{X}}{\bf{w}}-{\bf{y}}\Vert }_{2}^{2}+\lambda {\Vert {\bf{w}}\Vert }_{2},{\rm{s}}.{\rm{t}}.\,{\bf{Z}}{\bf{X}}{\bf{w}}=0,$$

where **Z** contains confounding variables as rows. Note that the equality constraint is linear with respect to the unknown parameter **w**, the solution remains convex, and the solution is guaranteed to be uncorrelated with the confounders. Additionally, the identified biomarkers are linear combinations of the input features.

Human randomized controlled experiments can either rarely be conducted in human etiological research, or are limited in examining one or a handful of causes at a time, posing a challenge of causal inference in psychiatric research. A wide range of computational causal discovery approaches have been developed to identify direct or indirect causal factors for clinical or neuropsychiatric outcomes, while accounting for unmeasured confounders [66–68].

### Critique #3: The Achilles heel of AI in psychiatry—limited interpretability and explainability

AI in psychiatry faces a major challenge: the “black-box” nature of its algorithms can obscure the reasoning behind predictions, complicating their clinical, legal, and social acceptability [41]. Understanding why an AI model predicts mental illness is crucial for fostering trust among psychiatrists, patients, and other stakeholders [42]. Transparent or “glass-box” models are urgently needed, as explainable AI (XAI) can help psychiatrists reconcile their intuition with AI-generated insights, enabling more trustworthy decision-making [43, 44]. However, balancing complexity and interpretability remains difficult; simple models (e.g., decision trees) offer better transparency, but complex structures (e.g., deep neural networks) often outperform them in predictive accuracy.

Explainability is also essential for patient engagement. Trust in treatment plans can diminish if patients are unable to understand AI-driven decisions. Tools, interfaces, and visualizations should make AI predictions accessible, tailoring explanations to individuals’ comprehension levels [45]. Despite growing interest in XAI, recent research suggests that interaction with AI recommendations does not consistently improve clinicians’ treatment accuracy—and incorrect recommendations may even mislead them [46].

Achieving deeper explainability requires addressing causality, not just correlation [47]. AI tools currently fall short of accounting for life events, behavioral feedback, and neural changes that influence mental health. A closed-loop framework combining neurostimulation with XAI could help uncover brain-behavior causation and drive precision treatments [16]. Until then, AI systems in psychiatry lack the transparency necessary for broad adoption and reliable use.

### Critique #4: Poor regulation of AI in mental health

AI-driven mental health tools, including digital phenotyping and chatbots, face significant regulatory gaps [48]. Social media platforms and mental health apps often self-regulate, leaving users, especially underage ones, vulnerable. This lack of oversight raises accountability concerns: Who is responsible when AI fails in mental health care? Insufficient regulation regarding ethics, fairness, data security, and explainability exacerbates the risks, favoring short-term benefits for industry players over long-term societal safety. Frameworks like the EU General Data Protection Regulation (GDPR) and the proposed European AI Act aim to tackle these issues, emphasizing transparency to build trust in AI tools [49].

Mental health chatbots, such as Woebot or ChatGPT, exemplify both the promise and pitfalls of AI in psychiatry. They offer scalability and 24/7 support, but their efficacy and safety remain under scrutiny [50]. Small-scale studies have faced criticism for overstating benefits and overlooking limitations like insufficient therapeutic relationships, data privacy risks, and questionable long-term trust. While such AI tools may augment care, they cannot yet replace psychiatrists, whose in-person connections remain the gold standard for psychiatric assessment. Until robust regulations and evidence emerge, applying AI to high-stakes mental health scenarios remains risky.

## Discussion and outlook

**Fig. 2:**
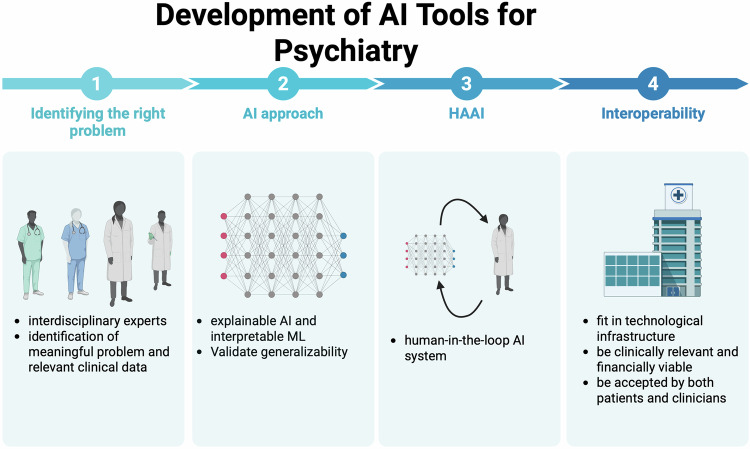
Schematic flowchart of developing practical and trustworthy AI tools for psychiatry. Step 1: Identify the right clinical problem and state clearly the goal and expectation of AI solutions. Step 2: Develop explainable AI or interpretable ML approaches. Step 3: Develop human-in-the-loop AI system by incorporating feedback from clinicians and end-users. Step 4: Maximize interoperability and clinical viability. (Created in and licensed by BioRender. https://BioRender.com/oy2zcmf).

Overall, the challenges of embedded bias, catastrophic interference, and cybersecurity vulnerabilities remain inherent in current AI systems. Furthermore, ethical concerns around patient privacy and data security cannot be ignored. Premature, widespread deployment of AI in mental health care could pose significant risks, especially without proper education for clinicians on interpreting AI results that diverge from established clinical decisions. To mitigate these pitfalls and enhance the safe integration of AI into psychiatry, we discuss several practical guidelines to enhance AI’s clinical viability (Fig. 2).

### Identifying the right problem and enhancing interoperability

Psychiatric disorders are highly complex and heterogeneous, not only in the manifestation but also in the etiology. Because the constructs are difficult to define, diagnose, and predict, this would require interdisciplinary collaboration to define clinically relevant problems for AI to target as well as to choose data collection in clinical practice. Clinicians, AI researchers, and end-users (providers and patients) must work together to identify meaningful predictors, outcomes, and data collection strategies aligned with clinical needs [51]. Selecting appropriate proxies and predictors, rooted in theoretical and practical relevance, is key.

For AI tools to be practical, they must fit within existing technological infrastructures. Factors integrated into the AI pipeline, such as fMRI or genetic data, must balance relevance and accessibility, as expensive or inaccessible methods are impractical for routine care. Wearable devices may streamline collection but risk limiting actionable outcomes compared to broadly accessible technologies. New AI approaches should leverage routinely collected data that clinicians can easily use at the point of care [52, 53]. To enhance interoperability, AI-assisted tools must be developed and validated on diverse, real-world samples under routine care conditions. Their efficacy and cost-effectiveness should be benchmarked against standard care practices to ensure meaningful clinical utility.

### Maximizing clinical viability and minimizing translational gap

While AI shows promise in psychiatry, improved prediction accuracy does not always translate into better clinical outcomes [54]. To enhance clinical utility, AI innovations must address clinical relevance, financial feasibility, and clinician acceptance [55]. Low-cost, scalable digital phenotypes are rapidly emerging but require validation to confirm whether they complement or improve upon traditional metrics. Demonstrating their clinical impact demands establishing robust associations between these digital phenotypes and established neuropsychological, neurobiological, behavioral, and psychometric measures. Rigorous evaluations, including randomized controlled trials and long-term studies, are essential for assessing their real-world effectiveness.

AI tools must integrate seamlessly into existing healthcare infrastructure to minimize financial and operational barriers. For example, incompatibility with EHR systems could significantly increase adoption and maintenance costs. Trustworthy AI systems are key to clinician acceptance [56], necessitating a re-examination of every step in the data pipeline—from initial design to cleaning, annotation, and evaluation. Data-centric AI approaches will play a critical role in this process [57]. Furthermore, involving clinicians and patients in the co-design of AI tools can improve trust, aligning these systems with practical clinical needs and ensuring their relevance in real-world settings.

### Integration of AI into clinical workflow

Integrating AI into clinical workflows requires more than just technical readiness—it demands organizational, educational, and cultural adaptation. The first step is to identify where AI can add tangible value without disrupting the clinician’s workflow—such as automating repetitive documentation, assisting with image analysis, or triaging cases based on risk. Successful implementation depends on co-designing tools with clinicians, ensuring that AI outputs are interpretable, transparent, and seamlessly integrated into existing electronic health record systems. Hospitals should establish AI governance committees to oversee data quality, regulatory compliance, and model validation in real-world settings. Pilot programs and iterative testing help fine-tune the system before broad deployment, allowing for feedback loops that improve both accuracy and usability

Equally critical is training the clinical workforce to understand and trust AI-assisted decisions. Educational modules—ranging from continuing medical education (CME) courses to simulation-based training—can help clinicians interpret AI outputs, recognize limitations, and integrate them into patient care. Building patient trust also requires clear communication: clinicians should explain how AI contributes to diagnosis or treatment while emphasizing that human oversight remains central. Public engagement, transparency about data use, and demonstration of improved outcomes will be key to patient acceptance.

### Human-assisted AI (HAAI) for psychiatry

AI models are prone to errors over time due to shifts in data distribution, user behavior, and clinical practices, which degrade their generalizability. To maximize the clinical relevance of AI in psychiatry, human-in-the-loop systems, a paradigm of augmented intelligence, offer a promising solution. These systems integrate continuous human feedback to address biases and inconsistencies, improving AI performance iteratively.

The idea of developing human-in-the-loop AI systems is not new in high-stakes decision-making processes [58] and has been advocated in mental health research [8, 59]. Clinicians can enhance AI models by providing incremental feedback, contributing prior knowledge, and introducing relevant data to refine decision-making and bolster system trust. Human intervention also enables top-down knowledge discovery and interactive learning [60] to improve the interpretability of AI outputs. This collaborative clinic-to-research feedback loop and human-AI interactions accelerate model adaptation and mitigate data biases.

In tandem with these efforts, merging data-driven AI approaches (uncovering patterns in psychiatric data) with theory-driven systems (modeling mental disorders as computational anomalies) can further advance psychiatric research [61, 62]. Integration of these two approaches will likely produce a fruitful outcome. Ultimately, fostering a collaborative environment where human expertise and AI capabilities complement each other will be essential for enhancing the effectiveness, reliability, and impact of mental health care.

### Data and software sharing initiatives

The exponential rise in AI research in psychiatry has led to numerous studies applying off-the-shelf algorithms to small datasets. However, these efforts often lack rigor and transparency, misleading readers and fostering skepticism among mental health professionals. To address this, researchers must prioritize transparent reporting, including clear discussions of AI limitations. Advancements in generative AI and LLMs offer transformative potential. For instance, “NYUTron,” trained on over 7 million clinical notes, showcases how fine-tuned models can predict health outcomes like hospital readmission. By continuously adjusting training data to mitigate biases, such systems could significantly impact mental health care [3].

For psychiatry to achieve the same breakthroughs as AI in computer vision and natural language processing, data and software sharing must become the norm. Open-access datasets, standardized benchmarks, and reproducible systems have been pivotal in other fields and should be widely adopted in psychiatry. Public repositories for de-identified psychiatric data—combined with government-supported frameworks for quality control, standardization, and regulation—are essential to drive innovation. Oncology biomarker research serves as a blueprint for coordinating multi-stakeholder efforts [63].

Additionally, community-driven competitions could incentivize the development of tools to detect AI biases in mental health, fostering the creation of robust “by-the-AI, for-the-AI” solutions. To ensure clinical relevance, validation criteria must be standardized, with benchmarks established using large, diverse datasets that account for factors like age, sex, ethnicity, and treatment history. Generalist medical AI (GMAI) applications could be leveraged to regulate and validate AI tools, ultimately advancing mental health care [64]. This strategic alignment of data sharing, benchmark creation, and interdisciplinary collaboration will be crucial for unlocking AI’s full potential in psychiatry.

### Recommendations

To close this discussion, we enclose several recommendations that directly address the individual critiques.

First, mitigating bias in AI-driven mental health research requires systematic bias auditing, diverse data representation, and transparent reporting. Datasets should encompass demographically and clinically heterogeneous populations, with rigorous validation to minimize underrepresentation and labeling errors. Embedding continuous bias monitoring into model training and deployment pipelines will enable adaptive correction over time. Close collaboration between clinicians and data scientists is crucial to align algorithmic outputs with clinical validity and ethical standards, thereby enhancing model fairness, generalizability, and reliability.

Second, advancing AI-based biomarker discovery demands methodological rigor and stronger alignment between biological and clinical data. Future studies should integrate multimodal datasets through standardized preprocessing pipelines, explicit handling of confounders, and prospective, hypothesis-driven designs. Moreover, linking biological signatures to longitudinal clinical outcomes through frameworks like the RDoC can enhance translational validity. Collaborative consortia and open data initiatives will be vital to achieve robust, reproducible, and clinically actionable biomarkers.

Third, improving interpretability in psychiatric AI necessitates the development of explainable and causally informed models. Integrating XAI frameworks that balance accuracy with transparency can help clinicians and patients understand and trust model predictions. Hybrid approaches combining mechanistic modeling, causal inference, and human-in-the-loop design offer a path toward interpretable yet high-performing systems, thereby strengthening both clinical decision-making and accountability.

Fourth, ensuring the safe and ethical deployment of AI in mental health requires stronger regulatory oversight. Future policies should enforce transparency, data protection, and accountability standards while differentiating between low- and high-risk applications. Multistakeholder collaboration among clinicians, ethicists, regulators, and technologists will be essential to establish evidence-based guidelines and certification pathways that promote responsible innovation and public trust in psychiatric AI.

## Conclusion

In conclusion, the journey to create trustworthy AI systems for mental health is filled with both promise and significant challenges. Like any successful partnership, the marriage between AI and psychiatry demands clear communication, mutual trust, patience, and persistent effort from all stakeholders. We stand at a crossroads where opportunities and obstacles coexist, but consensus across the involved parties remains elusive [65]. Our hope is that this commentary not only sparks critical dialogue but also inspires collaboration between AI researchers and AI-savvy clinicians. By addressing these issues with focused intent, we can restructure research strategies to deliver clinically meaningful outcomes. If this effort motivates a paradigm shift toward actionable and reliable solutions in mental health, we will have achieved our goal. The future of AI in psychiatry lies not just in possibilities, but in our collective commitment to making it a reality.
